# Combining Domestic and Foreign Investment to Expand Tuberculosis Control in China

**DOI:** 10.1371/journal.pmed.1000371

**Published:** 2010-11-23

**Authors:** Zhong-wei Jia, Shi-ming Cheng, Zhi-jun Li, Xin Du, Fei Huang, Xiao-wei Jia, Peng Kong, Yun-xi Liu, Wei Chen, Wei Wang, Christopher Dye

**Affiliations:** 1 National Institute on Drug Dependence, Peking University, Beijing, China; 2 National Center for Tuberculosis Control and Prevention, Chinese Center for Disease Control and Prevention, Beijing, China; 3 Colleges of Arts & Science of Beijing Union University, Beijing, China; 4 National Institute of Occupation Health and Poison Control, Chinese Center for Disease Control and Prevention, Beijing, China; 5 Foreign Loan Office, Ministry of Health P. R. China. World Bank Loan/UK Grant TB Control Project, Beijing, China; 6 Chinese PLA General Hospital 28, Beijing, China; 7 School of Public Health and Family Medicine, Capital Medical University, Beijing, China; 8 HIV/AIDS, Tuberculosis, Malaria and Neglected Tropical Diseases, World Health Organization, Geneva, Switzerland

## Abstract

Jia and colleagues describe how a combination of increased domestic funding, supplemented by foreign loans and donations since 2002, have led to a dramatic increase in tuberculosis case finding in China.

**Summary Points**

- Responding to evidence that many individuals with tuberculosis (TB) did not have access to treatment in 2001, especially in poorer areas of China, the government changed its policy on TB control. Domestic TB funding was increased nationwide, supported by a World Bank loan and donations from the Global Fund and other foreign agencies.
- Under the new funding initiative, case notifications more than doubled between 2002 and 2005, sufficient for China to reach the WHO targets of detecting 70% of all new smear-positive cases and successfully treating 85% of these cases nationwide.
- The cost of finding and successfully treating each case between 2003 and 2008 was estimated to be US$272 on average—a cost-effective policy by comparison with other health interventions.
- While changes in TB control policy produced clear benefits in case finding, they present two challenges for evaluation, concerning (1) the impact of better case finding on TB transmission, case load, and mortality, and (2) the contribution to TB control of system-wide improvements in health care initiated in 2003 and 2009.

China had an estimated 1.3 million new cases of tuberculosis (TB) in 2008, of which 112,000 were multidrug resistant. That is, nearly 0.1% of the Chinese population developed TB for the first time in 2008, accounting for one in seven new cases worldwide [1],[2]. Over the period 2001–2008, TB was the second largest cause of death among China's 39 notifiable communicable diseases, behind HIV/AIDS [3].

The Ministry of Health first began to scale up TB control in 1992 by adopting the World Health Organization (WHO) Directly Observed Treatment Short-course (DOTS) strategy, covering 13 of 31 provinces (about half the national population), assisted by a World Bank loan. DOTS, now enhanced as the Stop TB Strategy, advocates early diagnosis of cases with active TB (target 70% of all new sputum smear-positive cases to be detected), followed by combination chemotherapy to achieve high cure rates (target >85% treatment success) and reduce transmission [1],[4]. During the decade 1990–2000, this large project cut the number of TB deaths in China by one half (evaluated from routine surveillance data) and the prevalence (measured in cross-sectional surveys) by one third [5],[6]. But when external funding ended in the year 2000, efforts diminished and TB case notifications declined, especially in some World Bank project areas [7]. A special study attached to the year 2000 national prevalence survey highlighted some of the persistent problems in TB control. For instance, 92% of a sample of 1,278 patients were not covered by medical insurance, and had sought diagnosis and treatment at their own expense. Nearly half (45%) of these patients failed to complete treatment because costs were too high [7].

The inadequacies of TB control at the turn of the millennium were a stimulus to do much better. In 2002, two new funding initiatives were launched by the Chinese government to reinvigorate DOTS, and especially to improve TB control in poorer areas of the country [7]. One initiative was backed by a further loan from the World Bank, with additional support from other donors. The second was supported by a donation from the Global Fund. Underpinning this external funding, the Chinese Government—national, provincial, and in counties—increased expenditure on TB control nationwide from 2003 onwards [7].

Here, we show how this combination of increased domestic funding, supplemented by foreign loans and donations, led to a dramatic increase in TB case finding and, potentially, more effective reductions in TB burden. We also highlight the way in which changes in TB control policy, carried out in the wider context of health system reforms, generate challenges for monitoring and evaluation.

## Evaluating the Impact of New Funds for TB Control

We tracked the number of sputum smear-positive TB cases reported by China's 2,859 counties to the National TB Surveillance System (NTBSS) from 1 January 2001 to 31 December 2008 (Text S1). Counties were mapped geographically, divided into three groups according to the principal sources of funding (World Bank, Global Fund, Chinese Government), and classified according to income, expenditure, literacy, and ethnicity. The following analysis examines the link between domestic and foreign investment (excluding private out-of-pocket expenditure) and TB case notifications, in counties of different kinds and with different funding sources. Cost-effectiveness is judged from the perspective of the government as health care provider.

## Clusters and Hotspots of TB in China

**Figure 1:**
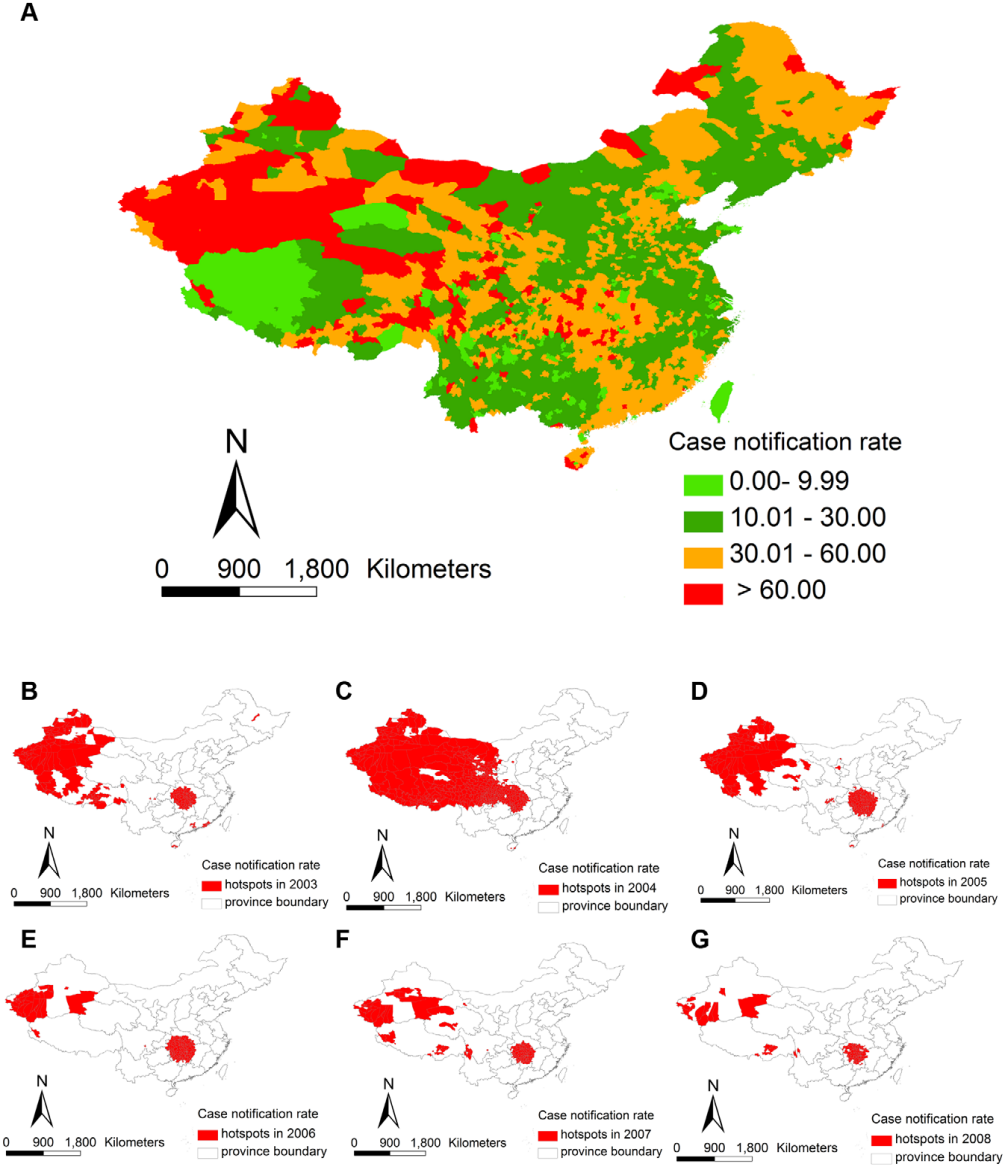
(A) Average TB case notifications per 100,000 populations by county, 2001–2008. (B–G) Distribution of counties that were TB hotspots over the period 2003–2008.

To decide how to allocate funds across China, the spatial distribution of TB cases was mapped to identify clusters (neighbouring counties with similar TB notification rates) and hotspots (counties with high notification rates within clusters) (Text S1). Mapping revealed that about 70% of the variation in the case notification rate was among provinces and 30% among counties (Text S1). Most of the variation was therefore on a larger (among provinces) rather than smaller (among counties) geographical scale (Figure 1A). However, there was significant clustering of cases at county level in each of the 6 years 2003–2008. Persistent hotspots were detected in western (Xinjiang, Neimeng, Gansu, Xizang, Qinghai, Shaanxi provinces) and central China (Hunan, Hubei, Henan, Chongqing, Guizhou provinces), and 307,638 cases were reported from these hotspots between 2003 and 2008, an average of 31 cases/100,000 population/year. Allowing for differences among counties in age and sex, case notification rates were higher in poorer counties, and in countries populated by minority groups (Text S1).

## Scaling Up TB Control

The resources provided by external donors, principally the World Bank and the Global Fund, were matched by funding from central and local government and used to support TB control in counties that are inhabited by more disadvantaged people (Table 1). Between 2003 and 2008, 81%–98% of hotspots were in World Bank and Global Fund areas (Figure 1B–1G). All aspects of TB control were improved from 2001 onwards: DOTS coverage increased from 44% of counties in 2001 to 100% in 2005; more staff were recruited and trained (1.7 million trained in 2005 alone); more laboratories were built (increasing in the World Bank area from 992 in 2002 to 1,479 in 2008); diagnostic evaluation became more intensive (1.7 million chest X-rays taken in World Bank in 2002, compared with 6.1 million in 2008); a greater number of TB cases was identified through routine health checks, as well as among those with symptoms typical of TB attending clinics; patient registration and clinical management were improved; a special fund was created for TB drugs, free treatment was extended to smear-negative patients; and health promotion was amplified through the various media. To achieve this, the Chinese government effectively combined domestic resources with those provided by foreign donors—in money and in kind (Text S1).

**Table 1:** Number and characteristics of counties in areas supported mainly by the World Bank, the Global Fund, and the central and local Governments.

Group	Total Number of Counties	Number of Counties in Income Groups (% counties)			Average County Expenditure, LCE CNY/Person	Average Level of Illiteracy in Counties, ILR%	Number of Minority Counties, MIN (% Counties)
		Poor	General	Developed			
WB	1,649	443 (27)	1,188 (72)	18 (1)	0.042	11	328 (20)
GF	1,331	580 (43.7)	747 (56)	4 (0.3)	0.049	19	326 (25)
CG	512	16 (3)	418 (81)	78 (16)	0.061	9	4 (0.8)

The classification of counties is described in Text S1.

CG, Chinese government; GF, Global Fund; ILR, illiteracy rate; LCE, per capita expenditure; MIN, minority county; WB, World Bank.

## Increasing Case Notification Rates

**Figure 2:**
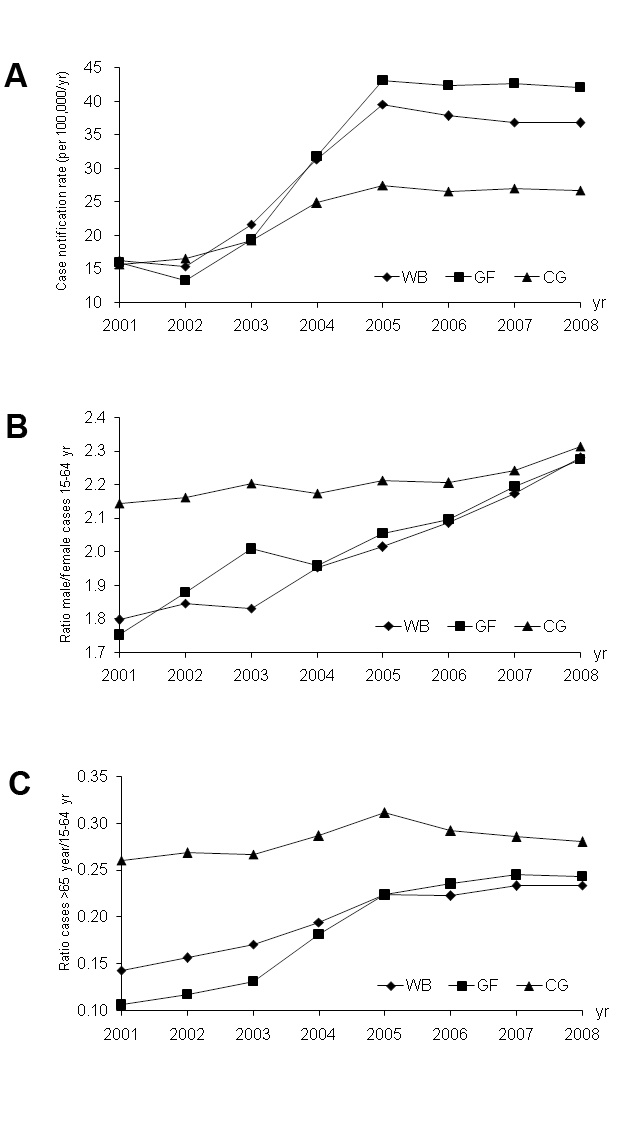
Changes in TB case notifications in different areas of China from 2001 to 2008. Trends in (A) TB case notification rates in areas supported by the World Bank (WB), the Global Fund (GF) and the Chinese government (CG); (B) the ratio of male/female cases; (C) the ratio of cases in people aged ≥65 years to cases in people aged 15–64 years.

During the 1990s, the number of TB cases (all forms) reported each year was typically between 30 and 40 per 100,000 populations, but, with further investment, began to increase in the early 2000s. A total of 2.9 million new sputum smear-positive cases were reported between 2001 and the end of 2008. The number of cases reported per capita increased in World Bank, Global Fund, and government-supported areas (Chinese government) from 2002 to 2005, and then stabilized or began to fall (Figure 2A; Table 2; Text S1). Compared with 2002, the increases by 2005 were much bigger in World Bank (236%) and Global Fund (224%) than in Chinese government areas (65%).

**Table 2:** Expenditures (2001–2005) and budgets (2006–2008) for TB control from different areas and funding sources.

Area of Country Identified by Main Source of Funding	Sources of Funding	Indicator	2001	2002	2003	2004	2005	2006	2007	2008	Total
**WB**	WB, UK Department for International Development, Japan International Cooperation Agency, Damien Foundation Belgium, central and local government funds matched to donors.	Expenditure or budget (CNY millions)	53.1	109.9	165.5	521.9	529.5	514.7	548.2	583.7	3,026.5
		Cost per person (CNY)	0.078	0.157	0.234	0.736	0.742	0.738	0.782	0.833	0.538
		Case notifications per 100,000 population	16.3	15.4	21.8	31.4	39.5	37.9	36.9	36.81	29.5
		Cost per TB case notified (CNY)	474	1,018	1,076	2,321	1,929	1,929	2,118	2,222	1,820
		Cost per TB case successfully treated (CNY)	496	1,065	1,124	2,457	2,053	2,052	2,262	2,380	1,930
		Incremental cost per TB case notified (CNY)	—	—	1,215	3,525	2,521	2,549	2,907	3,063	2,778^a^(2,696)
**GF**	GF, central and local government funds matched to donors.	Expenditure or budget (CNY millions)	18.7	22.9	116.1	162.9	177.7	181.9	202.9	175.2	1,058.3
		Cost per person (CNY)	0.077	0.095	0.475	0.663	0.721	0.766	0.855	0.722	0.546
		Case notifications per 100,000 population	15.9	13.3	19.5	31.85	43.1	42.4	42.7	42.1	31.3
		Cost per TB case notified (CNY)	482	711	2,350	2,057	1,732	1,819	2,046	1,713	1,763
		Cost per TB case successfully treated (CNY)	505	740	2,455	2,170	1,851	1,943	2,176	1,811	1,857
		Incremental cost per TB case notified (CNY)	—	—	5,452	2,986	2,202	2,348	2,692	2,175	2,594^a^ (2,890)
**CG**	Central and local government funding	Expenditure or budget (CNY millions)	61.2	89.4	101.4	174.8	197.6	165.3	202.9	120.2	1,112.8
		Cost per person (CNY)	0.187	0.271	0.304	0.519	0.580	0.464	0.559	0.330	0.404
		Case notifications per 100,000 population	15.7	16.6	19.3	24.9	27.5	26.6	27.1	26.8	23.2
		Cost per TB case notified (CNY)	1,187	1,637	1,588	2,090	2,046	1,819	2,191	1,233	1,763
		Cost per TB case successfully treated (CNY)	1,242	1,723	1,669	2,225	2,186	1,933	2,322	1,303	1,867
		Incremental cost per TB case (CNY)	—	—	1,299	2,930	2,578	2,093	2,996	2,556	2,556^a^ (2,560)
**National total**		Expenditure or budget (CNY millions)	133.0	222.3	382.9	860.6	904.9	861.9	954.0	879.1	5,197.6
		Cost per person (CNY)	0.106	0.174	0.298	0.665	0.696	0.667	0.734	0.672	0.504
		Case notifications per 100,000 population	16.0	15.3	20.6	29.8	36.9	35.6	35.3	35.0	28.2
		Cost per TB case notified (CNY)	655	1,140	1,434	2,217	1,914	1,883	2,117	1,901	1,794
		Cost per TB case successfully treated (CNY)	686	1,194	1,500	2,348	2,037	2,005	2,256	2,024	1,901
		Incremental cost per TB case notified (CNY)	—	—	2,230	33,04	2,449	2,434	2,864	2,455	2,639^a^ (2,724)

a These figures in the final column are averages over the period 2003–2008 (or 2003–2005 in brackets). Incremental costs are calculated in comparison with 2002.

CG, Chinese government; GF, Global Fund; WB, World Bank.

After 2001, more cases were found among both males and females, but the increase was greater among males, so that the ratio of male/female cases nationwide rose steadily from 2.1 to 2.5 over the 8-year period to 2008 (Text S1). The increase in the sex ratio was greater in World Bank and Global Fund areas as compared with Chinese government areas, rising from 1.9 to 2.5 in World Bank and Global Fund combined but only from 2.5 to 2.6 in the Chinese government. Within World Bank and Global Fund areas, the increase in sex ratio was predominantly in the 15–64-year age class, and the sex ratio in this age class had almost converged in World Bank, Global Fund, and Chinese government areas by 2008 (Figure 2B).

The intervention in World Bank and Global Fund areas also favoured elderly patients (>65 years), both males and females, in comparison with patients aged 0–14 years and 15–64 years. The ratio of older to younger adult cases increased throughout the period 2001–2008 in World Bank and Global Fund, but not markedly in the Chinese government (Figure 2C; Text S1).

The number of cases reported in children 0–14 years also increased in all three areas between 2002 (2,077 cases) and 2005 (3,282 cases), but this increase was smaller (27%) than for all adults (143%). Finally, in association with increases in notifications in all three areas, the proportion of smear-positive cases with recurrent TB after prior treatment dropped from 15.1% in 2003 to 8.6% in 2006, indicating that cure rates were improving.

## Expenditure, Cases Notified, and Cost-Effectiveness

The total government expenditure on TB control for the period 2001–2008 was CNY5,198 million (US$743 million) (Table 2; Text S1). Expenditures increased in all areas from 2001 to 2005, and budgets remained approximately stable from 2006 to 2008. So the number of cases reported from 2001 to 2008 has closely tracked spending on TB control. More than half of the national budget for TB control was allocated to the World Bank area in 2008.

The average cost per TB case notified over the whole period 2001–2008 was similar in World Bank, Global Fund, and Chinese government areas (CNY1,763–1,820, US$252–US$260). Approximately 94% of the 2.9 million new sputum smear-positive cases were reported to be successfully treated in each of the three areas of the country. Based on these treatment results, the cost per case successfully treated in the three areas over the period 2001–2008 was CNY1,857–1,930 (US$265–US$276; Text S1).

Assuming that the case notification rates of 2002 would have persisted from 2003 to 2005 without additional funding, 329,108, 134,219, and 80,712 additional cases were found in World Bank, Global Fund, and Chinese government areas over this 3-year period. The incremental costs for each extra case notified and put on treatment were greater than the total costs per case, but similar in all three areas despite differences in the numbers of additional cases reported. They were in the range CNY2,696–2,890 (US$385–US$413; Text S1).

## China's Changing Policy on TB Control: What We Know and Don't Know

China's approach to TB control since 2002 is a lesson in effectively managing domestic and foreign investment to improve health. By stepping up domestic funding for TB control, and adding in contributions from external agencies, the Chinese government more than doubled the number of cases reported each year. The biggest annual increases in funding were between 2002 and 2005, and this injection of funds apparently drove the large increases in TB cases reported over this period. This funding initiative has benefited children, young adults and the elderly, men and women, and TB cases living in all areas of the country. But the greatest benefits were for men (15–64 years), the elderly (≥65 years), and those living in the poorest counties, which were the focus of foreign donor support. The extra benefits to men, in particular, were not anticipated and remain to be explained.

From the perspective of the government as health provider, the average cost per case successfully treated was estimated to be CNY1,901 (US$272), which is a cost-effective policy on TB control by international standards. In studies carried out in Egypt, India, Malawi, Mozambique, Syria, Tanzania, and Thailand, also from the provider perspective, the costs per cure for outpatients treated by government health services ranged up to US$400, which is considered cost-effective among health interventions [8].

Although many of the benefits of investment are clear, China's changed policy on TB control leaves at least two key questions unanswered. Both are critical for future policy, but each presents a challenge for evaluation. First, it is not yet known how the increase in case finding has affected, and will affect, the transmission of infection, case load, and mortality. If China has exceeded the WHO targets of 70% case detection and 85% treatment success, then we would expect to see substantial reductions in transmission and in the number of cases and deaths. In this context, the findings of the 2010 national TB prevalence survey, now under way, will be crucial. The reduction in prevalence obtained over the past decade can be set against the investment in TB control over that period.

Second, while higher levels of funding for TB control were clearly associated with improved case notification, these new resources were provided at about the same time as China made other improvements to health services. Starting in 2003, the government progressively introduced three new health financing mechanisms: the rural cooperative medical scheme (insurance coverage was especially low in rural areas), the basic medical insurance system for uninsured urban residents (children, elderly people without pensions and the long-term unemployed, but not migrants), and the medical assistance programme (for those who receive the minimum living allowance) [9]–[11]. Those schemes have greatly extended the coverage of health care, although data on insurance coverage and on the utilization of township health facilities suggest that the main effects did not occur until after 2005 [9], by which time TB case notification rates had stabilized.

On the basis of our results up to 2008, we cannot yet anticipate the impact of China's latest health care reform plan announced in 2009 [12]. The new plan aims, among other things, to raise health insurance coverage still further, establish a national essential drugs system with price regulation and reimbursement, provide local medical care to reduce workloads in overstretched city hospitals, improve screening and prevention, and decommercialize public hospitals. New infrastructure will include thousands of township and county hospitals, compliant with national standards [9]. As China becomes more reliant on domestic rather than foreign investment, there is a premium on evaluating the links between financing for health in general and for TB control in particular. Studies of this kind are rare, and yet they are vital in setting future policy for the control of diseases like tuberculosis.

## Supporting Information

Alternative Language Summary S1 Chinese translation of the summary by ZJ and SC.
(0.03 MB DOC)

Text S1 Additional methods, models, and results.
(0.56 MB DOC)

## Abbreviations

DOTS: Directly Observed Treatment Short-course
TB: tuberculosis

## References

[1] World Health Organization (2009). Global Tuberculosis Control 2009: Epidemiology, strategy, finances..

[2] World Health Organization and Stop TB Partnership (2007). The Action Plan for Global MDR-TB and XDR-TB Control, 2007-2008..

[3] Ministry of Health P. R. China (2009). Report on status of national notifiable diseases in 2009. Ministry of Health P. R. China.. http://www.moh.gov.cn.

[4] Raviglione MC, Uplekar MW (2006). WHO's new Stop TB Strategy.. Lancet.

[5] Dye C, Zhao FZ, Scheele S, Williams B (2000). Evaluating the impact of tuberculosis control: number of deaths prevented by short-course chemotherapy in China.. Int J Epidemiol.

[6] China Tuberculosis Control Collaboration (2004). The effect of tuberculosis control in China.. Lancet.

[7] Wang L, Liu J, Chin DP (2007). Progress in tuberculosis control and the evolving public-health system in China.. Lancet.

[8] Floyd K, Arora VK, Murthy KJ, Lonnroth K, Singla N (2006). Cost and cost-effectiveness of PPM-DOTS for tuberculosis control: evidence from India.. Bull World Health Organ.

[9] Herd R, Hu YW, Koen V (2010). Improving China's health care system..

[10] Dong KY (2009). Medical insurance system evolution in China.. China Econ Rev.

[11] Chen Z (2007). New rural cooperative medical service will cover around whole country by 2008.. Xinhua 2007;.

[12] Chen Z (2009). Launch of the health-care reform plan in China.. Lancet.

