# Supplementary material for: Combining Domestic and Foreign Investment to Expand Tuberculosis Control in China
Source: PLoS Med. 2010 Nov 23;7(11):e1000371. doi: 10.1371/journal.pmed.1000371 (PMC2990708; doi:10.1371/journal.pmed.1000371)
Supplement: Alternative Language Summary S1 — Chinese translation of the summary by ZJ and SC. (0.03 MB DOC) [file pmed.1000371.s001.doc]

全新结核病融资模式对中国结核病防控的提升

成诗明Shiming Cheng，贾忠伟 Zhongwei Jia

2001年，在中国西北等贫困和边远地区有大量结核病人没有得到及时治疗。中国政府为此改变结核病防治政策，在全国建立一种全新的结核病融资模式，即加大各级政府结核病防治经费投入，同时引进外资支持，如世界银行贷款，英国赠款，全球基金等。

在全新结核病融资模式下，2002年至2005年之间，中国结核病人登记率增加一倍，并于2005年底实现了WHO提出的70%新涂阳病人发现和85%治愈的目标。这种新模式对在15-64岁的男性人群、65岁以上的老年人群以及贫困人群中发现额外的结核病人非常有效。

对2003-2008年完成治疗的新涂阳结核病人进行成本分析发现，中国发现并成功治疗1例结核病人的平均费用是272美圆（人民币1904元），与其它结核病卫生干预措施相比，符合成本效益。

但是，尽管中国在现有结核病防控策略下病人发现得到显著提高，若据此制订新的结核病防控政策还需进一步考虑几个关键问题：（1）大量病人发现对结核菌传播、现患人数和死亡率的影响；（2）中国实施的一系列卫生政策，如2003年启动的基础疫情报告系统和2009年的中国医疗改革，对结核病控制的影响。
