# Supplementary material for: Combining Domestic and Foreign Investment to Expand Tuberculosis Control in China
Source: PLoS Med. 2010 Nov 23;7(11):e1000371. doi: 10.1371/journal.pmed.1000371 (PMC2990708; doi:10.1371/journal.pmed.1000371)
Supplement: Text S1 — Additional methods, models, and results. (0.56 MB DOC) [file pmed.1000371.s002.doc]

**Combining Domestic and Foreign Investment to Expand Tuberculosis Control in China**

**Text S1: Additional Methods, Models and Results**

Zhong-wei Jia, Shi-ming Cheng, Zhi-jun Li, Xin Du, Fei Huang , Xiao-wei Jia, Peng Kong,Yun-xi Liu, Wei Chen, Wei Wang and Christopher Dye

The purpose of this study was to evaluate the effectiveness and cost-effectiveness of increased government funding (CG), supported by funds from external donors, mainly the World Bank (WB) and Global Fund (GF), in providing a high quality of care for TB patients in China. Patients who benefited from these funding schemes had access to diagnosis and treatment by known procedures and conforming to international standards. Other studies carried out in selected clinics and hospitals in various countries, have documented the cost-effectiveness of various components of TB control [1]. The present study, by contrast, assesses the effects of increased expenditure on the detection and treatment of TB cases through health services at national scale. Combining funding sources, the aim was to calculate the full costs and some important effects of large-scale expansion of a TB control programme.

**Definitions**

The following terms are used in this study:

**County** is a political subdivision of a province in China, which usually contains several townships and has a population of around 500,000 (mean 443,097, 95%CL 6540, range 2121– 6,958,904). China had a total of 31 provinces and 2859 administrative counties in 2008, excluding Taiwan, Hong Kong and Macau [2].

**GCE1** (**National** **Poorer Counties**): The Chinese Government has defined absolute poverty as an income of up to 683 CNY (US$ 98) and relative poverty as an income of up to 944 CNY (US$ 135) per person per year [3]. By weighing these indicators against average rural GDP and measures of income inequality such as the Gini-coefficient [4], 592 counties were identified as National Poorer Counties in 2005. 576 of these counties were covered by the two funding initiatives that began in 2002 (WB2 and GF) [5].

**GCE2** (**General Counties**): The other counties, ranked between the 592 National Poorer Counties (GCE1) and 100 Developed Counties (GCE3), are defined as General Counties.

**GCE3** (**Developed Counties**): Every year the National Bureau of Statistics of China recognizes the top 100 counties listed in the Comprehensive Economic Development Index as Developed Counties. The most recent yearbook used for this study was published in 2008 [6].

**LCE (Per Capita Expenditure)** refers to annual local government expenditure on public services per person [4, 6].

**ILR (Illiteracy Rate**) is the proportion of the population in any county that is illiterate, as defined in the China Statistical Yearbook [6].

**MIN** (**Minority County**) refers to whether a county is listed by the government as being inhabited mainly by minority groups, although people belonging to minorities are more widely distributed. China has a total of 658 Minority Counties, of which 257 were classified as National Poorer Counties (GCE1). The population of Minority Counties accounted for about 9% of the total Chinese population [6].

**DOTS (Directly Observed Treatment Short-course)**, the WHO-recommended TB control strategies, is watching the patient take his/her medication to ensure medications are taken in the right combination and for the correct duration. It combines five components: government commitment, case detection by sputum smear microscopy, standardized treatment regimen with directly observed treatment for at least the first two months, a regular drug supply, and a standardized recording and reporting system that allows assessment of treatment results.

**Study Design**

We classified funding for TB control into three parts according to the sources of funds and the geographical areas supported (Table S1, Figure S1):

(1) WB areas: although this detail is not included in the main text, WB areas are subdivided into WB1, WB2 and WB1+2 areas. WB2 is funded by the World Bank, the UK Department for International Development (DFID), the Japanese International Cooperation Agency (JICA) and the Damien Foundation of Belgium (DFB). The World Bank has supported TB control in China since 1991, so we also distinguished and compared those provinces that were supported during the first phase of Bank funding 1991-2000 (WB1), those supported during the second phase 2002-2010 (WB2), and those supported during both phases (WB1+2, Table S1). There was matched funding from central and local government in all phases. "WB" in the main text refers to WB2.

(2) GF area: supported by the Global Fund, with matched funding from central and local government.

(3) CG area: funded by the Chinese Government, national, provincial and at county level.


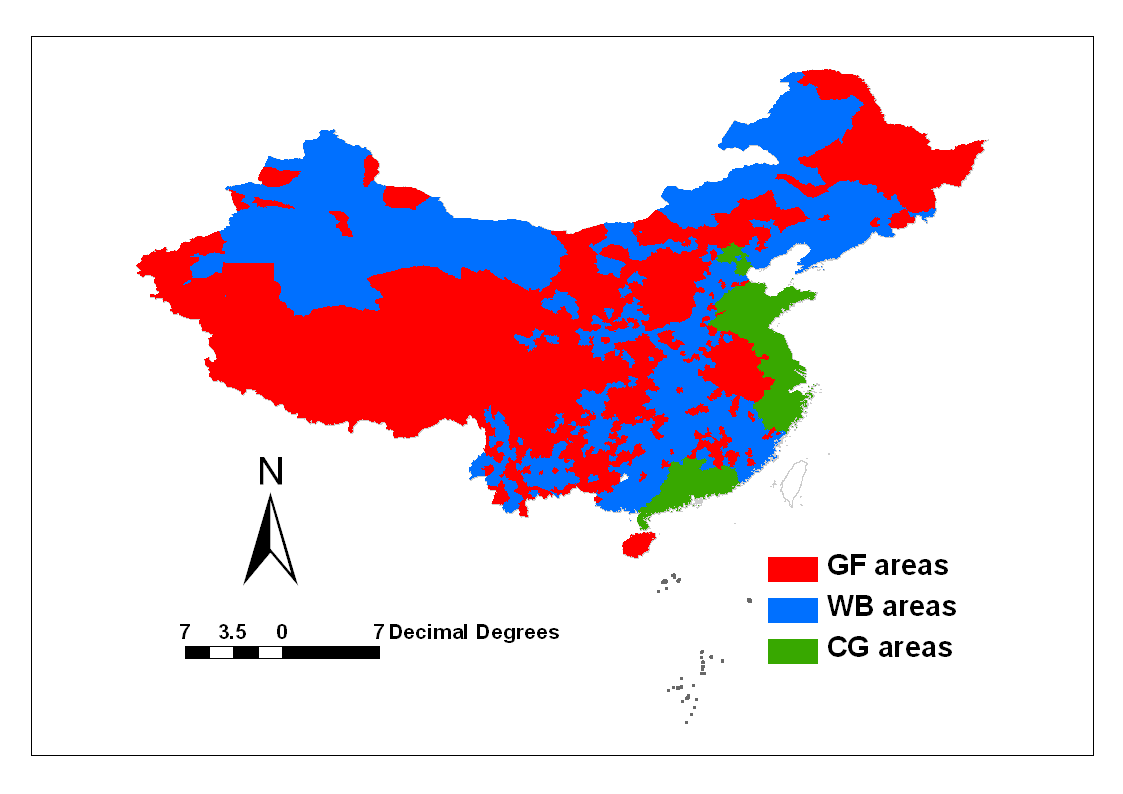


Figure S1. Geographical areas of China where TB control is supported mainly by the Global Fund (GF, red), the World Bank (WB, blue) and the Chinese government (CG, green)."

The funds from central government were specifically for TB control, and provided to 31 provinces based on the number of cases reported from each province. The necessary matched funding from provincial and county governments was also determined by numbers of reported cases. Our analysis is of government (CG) and government-led funding (WB2, GF), as distinct from private out-of-pocket expenditure.

The distribution of counties by income (GCE1-3), expenditure (LCE), illiteracy (ILR) and ethnicity (MIN) is shown in Table 1 of the main text. The counties in WB2 and GF areas were similar, whereas those in CG were inhabited by people with higher incomes and higher levels of literacy. In CG areas, local government expenditure was higher, and a small proportion of counties were inhabited by minorities. These statistics reflect the fact that support from the World Bank, the Global Fund and other external agencies was targeted at poorer and relatively disadvantaged people.

Compared with WB2 and GF areas, counties that received funds from local and central government only (CG area) tended to have wealthier and better educated populations, from which fewer additional cases were reported. CG areas covered the 7 wealthiest provinces in China, measured as GDP per capita. TB case notification rates in three of the metropolitan areas in CG (Beijing, Tianjin and Shanghai) have been the lowest nationwide (7-10 per 100,000 populations per year) since year 2000, and are similar to those in developed countries. And yet the cost and incremental cost per case reported were no greater in CG areas than in WB2 and GF areas. Further investigation is needed to explain this result, checking, for example, that there were no hidden costs at provincial or county level in CG areas.

**Data Management**

A total of 2,920,550 new sputum smear-positive TB cases from the National TB Surveillance System (NTBSS) were analyzed, covering 2859 counties in China from 1 January 2001 to 31 December 2008. NTBSS produces these data annually at county level in affiliation with the National Center for Tuberculosis Control and Prevention (NCTBCP) in China's Centers for Disease Control.

All TB cases were diagnosed according to standards issued by Ministry of Health of China, the latest in 2003. For a diagnosis of sputum smear-positive TB, a case must satisfy one of three sets of conditions: (a) 2 positive sputum smears by microscopy, (b) 1 positive sputum smear and 1 positive sputum culture, or (c) 1 positive sputum smear-positive with typical pathology of active TB on a chest X-ray [7].

The demographic data and literacy rates of each county were obtained from 2002-2008 censuses, provided by the National Bureau of Statistics of China. Per capita expenditure is defined in local currency and also converted to US$ at the 2008 exchange rate of CNY 7.0 per $US [6].

Map files of county boundaries were obtained from the Ministry of Water Resources. These were used to generate visual presentations at 1:100,000 scale with ArcGIS 9.1 software (ESRI Inc., Redlands, CA, USA).

**Spatial Statistical Analysis and Hotspots**

We mapped the distribution of cases (per capita) among counties using a GIS Spatial Model (Appendix) [8, 9]. The Global Moran statistic$I$ tests for spatial associations of disease at county level, where$I\in\left(-1,1\right)$. The value of $I$ depends on whether counties with similar TB notification rates are clustered ($I$ → 1), randomly distributed ($I$ = 0) or dispersed ($I$ → -1). To test whether cases were distributed non-randomly among counties, we used the standard normal deviate (*z*). When *z* > 1.96 and$p<0.05$, the TB case notification rate was considered to be clustered at the level of adjoining counties [8]. The Getis $G_{i}^{*}$statistic, also a measure of spatial autocorrelation (and using *z* scores) recognizes variation within patterns of spatial dependence. In this context, we used it to identify which counties were most likely to be hotspots within a cluster of similar counties. The larger the value of $G_{i}^{*}$ for any county *i*, the greater the influence of that county. Counties for which $G_{i}^{*}$ ≥ 1.96 and$p<0.05$ are taken to be hotspots for TB [9]. Because $G_{i}^{*}$identifies specific counties as hotspots, rather than simply clustering patterns, these counties can be mapped (Figure 1).

**Moran and Getis Statistics in Detail**

Here we give further details of the Moran and Getis statistics that are used to identify, respectively, clustering and hotspots of reported TB cases. Moran's $I$ is a weighted correlation coefficient used to detect departures of case notification rate from spatial randomness. It tests for spatial autocorrelation of the notifications rates from counties and determines whether the rates of neighboring counties are similar, measuring the spatial patterns of disease with $Z score$. Moran's $I$is defined by

$$I\left(d\right)=\frac{n\sum_{i}^{n} \sum_{j}^{n} w_{ij}\left(d\right)\cdot\left(y_{i}-\bar{y}\right)\left(y_{j}-\bar{y}\right)}{\left(\sum_{i}^{n} \sum_{j}^{n} w_{ij}\left(d\right)\right)\cdot\sum_{i}^{n} \left(y_{j}-\bar{y}\right)^{2}},$$

where $n=1,2...2386$ denote 2859 counties in our study; $y_{i}$ denotes TB case notification rate at county$i$; $\bar{y}$ is the mean value of $\left\{y_{i}\right\}$ over the 2859 counties; $w_{ij}$is the spatial weight measure defined as 1 if county $i$ is contiguous to county$j$ and 0 otherwise; $I\in\left(-1,1\right)$, where a positive value means that nearby counties have similar case notification rates indicating global spatial clustering and negative values indicate that nearby counties have different rates indicating global dispersion.

The $Z score$ are used to test the significance of $I$ values, which are used to determine the spatial pattern of disease. At a significance level of $p<0.05$, $Z score$ would have to be less than –1.96 or greater than 1.96, whereupon county case notification rates are significantly dispersed or clustered nationwide. The $Z score$ is defined by:

$$Z score\left(I\right)=\frac{I-E\left(I\right)}{\left[Var\left(I\right)\right]^{1/2}},$$

Getis’ $G_{i}^{*}$ statistic measures local spatial autocorrelation and is used to detect counties that are hotspots, among all 2859 counties. Getis’ $G_{i}^{*}$ is defined as:

$$G_{i}^{*}=\frac{\sum_{j}^{n} w_{ij}\left(d\right)\cdot y_{i}-W_{i}^{*}\cdot\bar{y}}{S\cdot\left\{\left[nS_{1i}^{*}-W_{i}^{*2}\right]/\left(n-1\right)\right\}^{1/2}},$$

where$n$, $y_{i}$, $\bar{y}$, are as above; $d$ is the threshold value of distance for a hotspot area; $S$ is the standard variance; $\left\{w_{ij}\right\}$is a symmetric binary spatial weighting matrix with$w_{ij}$ = 1 if county$j$is within a given distance $d$ from $i$ and 0 otherwise; $W_{i}^{*}=\sum_{j\neq i}^{n} w_{ij},$ $S_{1i}^{*}=\sum_{j\neq i}^{n} w_{_{ij}}^{2}.$ The higher the value of $G_{i}^{*}$ for county $i$, the greater the influence of that county. The counties with higher rates, which $G_{i}^{*}\geq1.96$, also a $Z score$, are defined as hotspots.

**Repeat Measures Analysis of Variance**

Repeat Measures Analysis of Variance (ANOVA) and Multilevel Extra-Poisson Regression Modelling with random and fixed effects (Models 1-3 below) were used to assess factors that affect case notification rates among different groups and through time. We monitored the change in TB case notification rates, nationally and in WB2, GF and CG areas, over the 8 years 2001­-2008. The analysis was done with SPSS (version 13.0) and SAS software (version 9.1). Significance tests were carried out with the$Wald\chi^{2}$statistic, taking a two-tailed$p$value of < 0.05 to be statistically significant.

**Multilevel Regression Models**

Three further statistical models were used to explore the way the number of smear-positive cases per 100,000 population notified by counties, expressed as logarithms, varied by (1) spatial scale, (2) poverty, literacy and ethnicity, and (3) age, sex and year (Appendix). To examine the spatial scale of variations in the case notification rate, we used a three-level model (model 1) where the province $v$ is at level 3, the county $\mu$ is at level 2 and the measurements repeated across years are at level 1 [18]. Model 1 was used to calculate the proportion of the variation in TB case notification rates that occurs among provinces $\sigma_{v0}^{2}$/ ($\sigma_{v0}^{2}$+$\sigma_{\mu0}^{2}$) and among counties $\sigma_{u0}^{2}$/ ($\sigma_{v0}^{2}$+$\sigma_{\mu0}^{2}$). Allowing for the effects of age (groups 0-14, 15-64, and ≥ 65 years) and sex, model 2 investigated whether changes in the county case notification rates between the minimum in 2002 and the maximum in 2005 were affected by income (GCE2 and GCE3 compared with GCE1), local government expenditure (LCE), illiteracy (ILR) and ethnicity (MIN). Model 3 assessed the variation of TB case notification rates among age groups, and by sex. We also investigated whether the funding stimulus had different effects on the case notification rate, by age (0-14 and 15-64 years compared with ≥ 65 years), sex (male compared with female) and across years. These different effects were evaluated statistically by examining the interactions between area, age group, gender and year.

**Structure of the Regression Models**

In order to test for factors that affect the variation in case notification rates (expressed in logarithms) in counties and provinces, we established 3-level models with provinces at level 3 ($k$), counties at level 2 ($j$) and repeated observations across years are at level 1 (i) (Tables S2 and S3). $\sigma_{v0}^{2}$ is the variance among provinces and $\sigma_{\mu0}^{2}$ is variance among counties. $A1_{ijk}$ is observed number of cases treated as a Poisson variate, and $\pi_{ijk}$is expected number of cases. The $offset_{ijk}=\log(pop_{ijk})$ensures the model is fitted to the case notification rate rather than to the number of cases, where $pop_{ijk}$ is population of county j in year i and in province k.

Model 1: case notification rate by county and province

Model 1 was used mainly to calculate the proportion of the variation in TB case notification rates that occurs among provinces $\sigma_{v0}^{2}/\left(\sigma_{v0}^{2}+\sigma_{\mu0}^{2}\right)$ and among counties$\sigma_{v0}^{2}/\left(\sigma_{v0}^{2}+\sigma_{\mu0}^{2}\right)$, where the two proportions sum to 1:


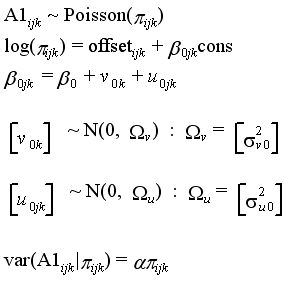


Model 2: case notification by poverty, literacy and ethnicity

Allowing for the effects of age (groups 0-14, 15-64, and ≥ 65 years) and sex, model 2 investigated whether changes in the county case notification rates between the minimum in 2002 and the maximum in 2005 were affected by income (GCE2 and GCE3 compared with GCE1), local government expenditure (LCE), illiteracy (ILR) and ethnicity (MIN):


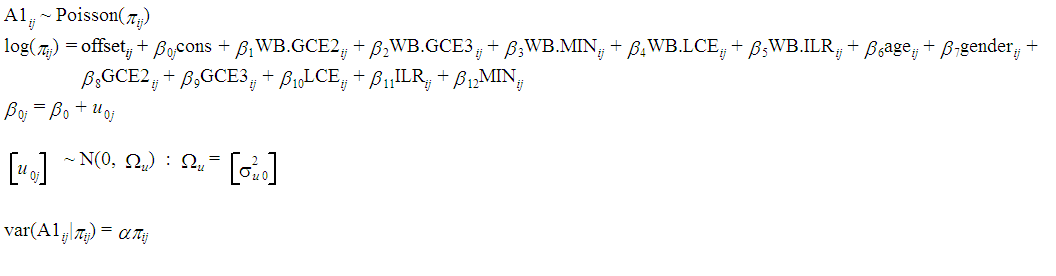


Model 3: case notification by age, sex and year

Model 3 assessed the variation of TB case notification rates among age groups, and by sex. We also investigated whether the funding stimulus had different effects on the case notification rate, by age (0-14 and 15-64 years compared with ≥ 65 years), sex (male compared with female) across years:

**Allocating Funds for TB Control**

As noted in the main text, all aspects of TB control were improved from 2001 onwards. To achieve this, the Chinese government effectively combined resources provided by various donors -- in money and in kind. Thus, JICA donated microscopes and drugs, and materials to support public educational programmes. DFB gave money to support case finding (including health promotion), diagnosis (including laboratory equipment), treatment (including drugs and case management), stock management, quality control, reporting procedures, training, surveillance, and evaluation. CIDA provided funds to train TB control staff at county level, and doctors in villages and hospitals; and to support case finding (including fees as incentives for heath workers), clinical case management, health promotion, and monitoring and evaluation. The Global Fund grant was given for drugs, equipment (diagnostic tests, microscopes, X-ray machines, computers, and vehicles), staff training, programme and patient management, and health promotion (highlighting drug resistance and TB among migrants).

A complete breakdown of costs, by item and donor, is not available. However, between 2001 and 2005, the following items of equipment were purchased with funds from named donors (in addition to any equipment given):

| Source of funds | Microscopes | X-ray machines | Vehicles | Computers | Ventilation cabinets | Film viewers |
| --- | --- | --- | --- | --- | --- | --- |
| CG | 0 | 0 | 0 | 0 | 2000 | 2000 |
| WB | 754 | 934 | 632 | 1292 | 0 | 0 |
| GF | 480 | 280 | 353 | 2256 | 0 | 0 |
| DFB | 18 | 1 | 26 | 11 | 65 | 0 |
| JICA | 1876 | 0 | 0 | 0 | 0 | 0 |
| Total | 3128 | 1215 | 1011 | 3559 | 2065 | 2000 |

**Further Analysis of Clusters and Hotspots**

As stated in the main text, there were significant clusters of TB cases at province and county level (Table S2). Two persistent hotspots were detected in north-western (Xinjiang, Neimeng, Gansu, Xizang, Qinghai, Shaanxi) and central China (Hunan, Hunan, Chongqing, Guizhou). Over 5 years, 81% (2003), 84% (2004), 93% (2005), 98% (2006) and 96% (2007) were covered by WB and GF areas.

**Further Analysis of Effectiveness of the Funding Programme**

TB case notification rates reached a peak in 2005. Compared with 2002, the increases by 2005 were 236% in WB, 224% in GF and 65% in CG (Fig 2A, main text). Repeat measures ANOVA showed that the interaction between funding scheme and year was significant (labeled year*WB2 in Table S2). Within-year comparisons of the interventions from 2003 onwards showed that case notification rates in WB and GF were markedly higher than in CG, but that WB and GF were not different from each other (Table S3A). Comparisons of each intervention in consecutive years (2002-2003, 2003-2004 etc) showed that case notifications increased significantly year on year in WB and GF but not in CG (Table S3B).

In comparisons of the areas that had been supported by World Bank funding for various periods since 1990, the bigger increases in case notification between 2002 and 2005 were in WB2 (163%) and WB1+2 (252%) rather than WB1 (35%), that is, in the areas that received further funds from 2002 onwards. Repeat measures ANOVA showed a significant interaction between funding scheme and year $\left(F=2.66, p<0.01\right)$. WB2 and WB1+2 groups showed striking increases between consecutive years, except WB2 between 2004 and 2005. Case notifications increased more gradually in WB1. Thus, new funding yielded a marked improvement in case finding even in the areas that had been supported during the 1990s [10, 11]. The implication is that sustained, adequate and targeted funding for TB control is vital to maintain high rates of case notification.

From 2002 to 2005, the increase in notified cases was greater among elderly people (≥65 years) than among children (0-14 years) and younger adults (15-64 years), and the effect was similar in WB (289% increase) and all other areas (266% increase). The increase in WB compared with all other areas was significantly greater for men (262% vs 65%) than for women (226% vs 59%) (2-way and 3-way year, gender and age interactions in Table S2). Case notification rates were also higher in poorer and minority counties (Table S2).

We do not know if still higher levels of expenditure from 2006-2008 would have yielded further increases in the number of reported cases. This is because the true incidence of TB in China has not been, and cannot easily be, measured with accuracy [12]. For the same reason, we do not know whether the disproportionate increases in cases found among men led, by 2005, to higher case detection rates (proportion of all true cases notified) among men than among women; we do not know the case detection rate among the elderly as compared with younger adults; and we do not know whether the apparent decline in the case notification rate after 2005 represents a real decline in incidence. Nevertheless, best estimates by WHO suggest that China had exceeded the international target of 70% case detection by 2005 [12].

Finally, there are some important outstanding questions about clinical outcomes and case notifications to do with, for example, the accuracy of reported treatment outcomes (>90% cure is high), and the reason why relatively few cases were found among children in 2008.

**Further Analysis of Cost and Cost-Effectiveness of the Funding Programme**

Although we have highlighted differences among geographical areas, the funding initiative that began in 2001 improved case notifications nationwide. And while the contributions of external donors have been important, the government of China provided the majority of the funds for TB control in WB areas (59%) and nationwide (70%) over the period 2002-2008. Between 2001 and 2008, the central government increased funding for TB control by a factor of 12, and provincial and county governments increased their contributions 2-9 fold. In 2007, the budget for TB control was at its highest level ever in China, exceeding CNY 950m (US$ 136m), and was only a little less in 2008 (CNY 879m, US$ 126m).

This analysis has been done from the perspective of the government as provider. Expenditure on TB control up to 2005 included the costs of diagnosis and treatment for new and relapsed smear-positive cases, plus cases with severe (cavitary and miliary) smear-negative disease. However the great majority of these cases (77% in 2005) were new smear-positive and our analysis of cost-effectiveness compares expenditures with the diagnosis and treatment of new smear-positive cases only.

Using recorded expenditures for the period 2002-2005 and budgets for 2006-2008 (expenditures for these years were not known at the time of analysis) we calculated the cost, and the incremental cost, of finding and treating new smear-positive cases in WB2, GF and CG areas. The budgets in 2006-2008 are likely to be a good substitute for expenditures in these years, given that budgets were almost completely spent between 2002 and 2005: 100% of central government budgets and 84% of local government budgets for TB control were reportedly spent over this period.

The cost per new smear-positive case diagnosed and put on treatment is the expenditure (2003-2005) or budget (2006-2008) in any year divided by the number of cases reported in that year. The cost per case successfully treated is the expenditure or budget divided by the number of cases cured (sputum smear became negative for acid fast bacilli by microscopy) plus those who completed treatment.

Incremental costs were calculated for each year 2003-2005 (expenditures) or 2006-2008 (budgets) as compared with 2002. They were also calculated as the annual average over the periods 2003-2005 (expenditures), or 2003-2008 (a combination of expenditures and budgets) as compared with 2002. The extra cases detected were the new smear-positive patients notified in each of the years 2003-2008 compared with 2002, or the annual averages for the periods 2003-2005 or 2003-2008 as compared with 2002. For instance, the incremental cost per new smear-positive case diagnosed and put on treatment in 2003 as compared with 2002 is the difference in expenditures for the two years divided by the difference in the number of reported cases. We cannot calculate the incremental costs per case successfully treated because we do not know treatment outcomes for patients not previously recorded.

Our national estimate of the absolute cost per case successfully treated (US$ 272) is approximate: the true value would be smaller because the money spent on TB control buys a wider range of services than the diagnosis and treatment of new smear-positive cases. This includes the treatment of smear-negative cases, which were approximately one third of all cases detected in 2003-2005 and around half of all cases detected in 2006-2008. On the other hand, US$ 272 is an underestimate if cure rates have been overestimated or if TB control has been supported by the expansion of health insurance coverage in rural areas and by the new rural cooperative medical service [13, 14].

**Table S1. Sources of funding for TB control in China. Figures are budgets for the stated project periods; some projects were not complete by 2008, and some expenditure was less than budgeted between 2001 and 2005.**

| **Source of funds** | **Study period** | **Provinces covered** | **Funding** | **Target** |
| --- | --- | --- | --- | --- |
| World Bank (WB1) | 1991-2000 | All counties in 7 provinces: Chongqing, Gansu, Hebei, Hubei, Hunan, Liaoning, Xinjiang, plus 6 provinces that were monitored during phase 2 (2002-2008, WB2) but received no additional funds: Guangdong, Hainan, Heilongjiang, Ningxia, Shandong, Sichuang. | Total funding CNY 1.067 bn, with CNY 0.481 bn from World Bank, matched by CNY 0.586 bn from central government. | Support activities of the National TB Control Institute, and reduce TB incidence, prevalence and mortality in 13 provinces. |
| World Bank/JICA/ DFID/DFB (WB2) | 2002-2008 | 9 provinces funded during phase 2 (WB2): Fujian, Guangxi, Guizhou, Henan, Inner Mongolia, Jiangxi, Jilin, Shanxi, Yunnan. 7 provinces funded by the World Bank during phases 1 and 2 (WB1+2): Chongqing, Gansu, Hebei, Hubei, Hunan, Liaoning and Xinjiang. The total of 16 provinces included 1649 counties (including 443 National Poorer Counties in GF areas) with 56% of the Chinese population. | Total funding CNY 2.011 bn, with CNY 0.863 bn from World Bank, CNY 0.203 bn from Central government, plus CNY 0.945 bn matched funding (CNY 0.829 bn from local government and CNY 0.116 bn from Japan International Cooperation Agency and the Damien Foundation Belgium). CNY 0.3 bn from DFID (UK) compensated for the interest on the World Bank loan. | Support all programme activities, with specific targets:  (1) Find and treat 2 million infectious cases during 2002-2008.  (2) More than 95% population in project areas will be covered by DOTS in 2008.  (3) Case detection rate for positive cases will reach 70% in 2008.  (4) Cure rate for positive cases will reach 85%. |
| Global Fund (GF) | 2003-2008 | 443 national poorer counties (GCE1) in 16 provinces: Chongqing, Fujian, Gansu, Guangxi, Guizhou, Hebei, Henan, Hubei, Hunan, Inner Mongolia, Jiangxi, Jilin, Liaoning, Shanxi, Yunnan and Xinjiang, and all counties in 8 provinces: Anhui, Hainan, Heilongjiang, Ningxia, Qinghai, Shanxi, Sichuang, Xizang. A total of 1331counties in 24 provinces covering 38% of the Chinese population. | Total funding CNY 1.113 bn, with CNY 0.931 bn from the Global Fund and CNY 0.182 bn from China government. | Provide diagnosis, free drugs, equipment, staff, training, health education, programme management; fund research, sociological evaluation. Reduce TB incidence, prevalence and mortality in 24 provinces. |
| Central government funding (CG) | 2002-2008 | All countries in all 31 provinces nationwide. | Total funding CNY 2.14 bn. | Funding allocated in proportion to the number of TB cases reported in the preceding year. |

**Table S2. Factors affecting TB case notifications, 2001-2008.**

| **Parameters** | **Estimate** | **Estimate（95%CI）** | **S.E** | **χ^2^ value** | **P value** |
| --- | --- | --- | --- | --- | --- |
| **Model 1** |  |  |  |  |  |
| **Fixed Effects** |  |  |  |  |  |
| Constant | -9.562 | (-9.717, -9.407) | 0.079 | 14656 | < 0.00001 |
| **Random Effects** |  |  |  |  |  |
| Level 3 $\sigma_{v0}^{2}$ (province) | 0.186 | (0.080, 0.282) | 0.049 | 14.4 | <0.0001 |
| Level 2 $\sigma_{\mu0}^{2}$ (county) | 0.081 | (0.073, 0.089) | 0.004 | 410.1 | <0.00001 |
| **Model 2** |  |  |  |  |  |
| **Fixed Effects** |  |  |  |  |  |
| Constant | -9.441 | (-10.55, -8.328) | 0.568 | 276.2 | <0.00001 |
| Age | -1.086 | (-0.545, -1.627) | 0.276 | 15.5 | <0.00001 |
| Gender | 1.562 | (0.915, 2.209 ) | 0.330 | 22.4 | <0.00001 |
| GCE2 (General) | -0.159 | (-0.108, -0.210) | 0.026 | 37.6 | <0.00001 |
| GCE3 (Developed) | 0.046 | (-0.025, 0.117) | 0.036 | 1.6 | =0.21 |
| LCE (Local Expenditure) | 3.102 | (2.553, 3.651) | 0.280 | 122.7 | <0.00001 |
| ILR (Illiteracy) | -0.003 | (-0.005, -0.001) | 0.001 | 9.0 | <0.005 |
| MIN (Minorities) | 0.101 | (0.046, 0.156) | 0.028 | 13.1 | <0.0005 |
| LCE*WB2 | 4.681 | (3.609, 5.751) | 0.547 | 73.2 | <0.0000 |
| ILR*WB2 | 0.001 | (-0.003, 0.005) | 0.002 | 0.25 | =0.61 |
| MIN*WB2 | -0.497 | (-0.642, -0.352) | 0.074 | 45.1 | <0.00001 |
| **Random Effects** |  |  |  |  |  |
| $\sigma_{\mu0}^{2}$ | 0.177 | (0.163, 0.191) | 0.007 | 639.4 | <0.00001 |
| **Model 3** |  |  |  |  |  |
| **Fixed Effects** |  |  |  |  |  |
| Constant | -8.502 | (-8.663, -8.341) | 0.082 | 10704 | <0.00001 |
| Year | 0.078 | (0.043, 0.113) | 0.018 | 18. 8 | <0.00001 |
| WB2 | 0.278 | (0.119, 0.437) | 0.081 | 11. 8 | <0.0001 |
| Age1 (0-14) | -0.475 | (-0.620, -0.330) | 0.074 | 41.2 | <0.00001 |
| Age2 (15-64) | -3.565 | (-4.096, -3.034) | 0.271 | 173.05 | <0.00001 |
| Gender | 0.739 | (0.617, 0.861) | 0.062 | 142.1 | <0.00001 |
| Age*WB2 | -0.159 | (-0.367, 0.049) | 0.106 | 2.25 | =0.13 |
| Gender*WB2 | -0.081 | (-0.183, 0.021) | 0.052 | 2.43 | =0.11 |
| Year*WB2 | 0.042 | (0.011, 0.073) | 0.016 | 5.3 | <0.01 |
| Age*gender | 0.645 | (0.404, 0.886) | 0.123 | 27.5 | <0.00001 |
| Year*age | 0.048 | (0.003, 0.093) | 0.023 | 4.6 | <0.05 |
| Year*gender | 0.022 | (0.004, 0.04) | 0.009 | 25.69 | <0.01 |
| Age*gender*WB2 | 0.116 | (-0.117, 0.349) | 0.119 | 0.95 | =0.33 |
| Gender*year*WB2 | 0.011 | (-0.030, 0.052) | 0.021 | 0.27 | =0.60 |
| Age*year*WB2 | 0.004 | (-0.023, 0.031) | 0.014 | 0.08 | =0.78 |
| Year*gender*age1 (0-14) | -0.061 | (-0.024, -0.098) | 0.019 | 10.3 | <0.005 |
| Year*gender*age2 (15-64) | -0.263 | (-0.108, -0.418) | 0.079 | 11.1 | <0.0005 |
| **Random Effects** |  |  |  |  |  |
| $\sigma_{\mu0}^{2}$ | 0.152 | (0.074, 0.230) | 0.040 | 14.4 | <0.0005 |

Note: * denotes interaction between two main effects

$\sigma_{v0}^{2}$ is the variance among provinces

$\sigma_{\mu0}^{2}$ is the variance among counties

**Table S3A. Analyses of variance comparing case notification rates within each year.**

|  |  |  | **F** | **p-value** |
| --- | --- | --- | --- | --- |
| **Time WB2, GF, CG groups** | | | | |
| 2001 year | WB2 vs. GF | | 2.070 | 0.152 |
|  | WB2 vs. CG | | 3.640 | 0.058 |
|  | GF vs. CG | | 8.260 | 0.005 |
| 2002 year | WB2 vs. GF | | 1.450 | 0.230 |
|  | WB2 vs. CG | | 0.870 | 0.352 |
|  | GF vs. CG | | 3.330 | 0.069 |
| 2003 year | WB2 vs. GF | | 0.960 | 0.329 |
|  | WB2 vs. CG | | 8.440 | 0.004 |
|  | GF vs. CG | | 11.310 | 0.001 |
| 2004 year | WB2 vs. GF | | 4.820 | 0.029 |
|  | WB2 vs. CG | | 20.000 | 0.000 |
|  | GF vs. CG | | 33.100 | 0.000 |
| 2005 year | WB2 vs. GF | | 5.510 | 0.020 |
|  | WB2 vs. CG | | 52.000 | 0.000 |
|  | GF vs. CG | | 68.530 | 0.000 |
| 2006 year | WB2 vs. GF | | 4.520 | 0.034 |
|  | WB2 vs. CG | | 58.400 | 0.000 |
|  | GF vs. CG | | 71.750 | 0.000 |
| 2007 year | WB2 vs. GF | | 3.860 | 0.051 |
|  | WB2 vs. CG | | 51.120 | 0.000 |
|  | GF vs. CG | | 62.470 | 0.000 |
| **Time WB1+2, WB1, WB2 groups** | | | | |
| 2001 year | WB1+2 vs. WB2 | | 27.880 | 0.000 |
|  | WB1+2 vs. WB1 | | 0.260 | 0.607 |
|  | WB2 vs. WB1 | | 20.300 | 0.000 |
| 2002 year | WB1+2 vs. WB2 | | 18.400 | 0.000 |
|  | WB1+2 vs. WB1 | | 10.090 | 0.035 |
|  | WB2 vs. WB1 | | 14.330 | 0.000 |
| 2003 year | WB1+2vs. WB2 | | 37.370 | 0.000 |
|  | WB1+2 vs. WB1 | | 17.170 | 0.000 |
|  | WB2 vs. WB1 | | 22.140 | 0.000 |
| 2004 year | WB1+2 vs. WB2 | | 41.800 | 0.000 |
|  | WB1+2 vs. WB1 | | 12.740 | 0.000 |
|  | WB2 vs. WB1 | | 9.660 | 0.013 |
| 2005 year | WB1+2 vs. WB2 | | 12.440 | 0.000 |
|  | WB1+2 vs. WB1 | | 36.200 | 0.000 |
|  | WB2 vs. WB1 | | 19.460 | 0.000 |
| 2006 year | WB1+2 vs. WB2 | | 3.090 | 0.081 |
|  | WB1+2vs. WB1 | | 35.640 | 0.000 |
|  | WB2 vs. WB1 | | 22.140 | 0.000 |
| 2007 year | WB1+2 vs. WB2 | | 2.610 | 0.109 |
|  | WB1+2 vs. WB1 | | 17.400 | 0.000 |
|  | WB2 vs. WB1 | | 21.700 | 0.000 |

**Table S3B. Analyses of variance comparing case notification rates between successive years.**

|  | | **WB2+GF** | | **CG** | |
| --- | --- | --- | --- | --- | --- |
| **Time I** | **Time J** | **S.E** | **p-value** | **S.E** | **p-value** |
| 2001 year | 2002 year | 0.801 | 0.056 | 0.560 | 0.081 |
|  | 2003 year | 1.155 | 0.000 | 1.106 | 0.047 |
|  | 2004 year | 1.531 | 0.000 | 2.525 | 0.036 |
|  | 2005 year | 1.854 | 0.000 | 3.488 | 0.047 |
|  | 2006 year | 1.938 | 0.000 | 3.510 | 0.119 |
|  | 2007 year | 1.846 | 0.000 | 3.304 | 0.095 |
| 2002 year | 2001 year | 0.801 | 0.056 | 0.560 | 0.081 |
|  | 2003 year | 1.092 | 0.000 | 0.833 | 0.107 |
|  | 2004 year | 1.483 | 0.000 | 2.159 | 0.040 |
|  | 2005 year | 1.736 | 0.000 | 3.309 | 0.064 |
|  | 2006 year | 1.914 | 0.000 | 3.343 | 0.170 |
|  | 2007 year | 1.807 | 0.000 | 3.129 | 0.137 |
| 2003 year | 2001 year | 1.155 | 0.000 | 1.106 | 0.047 |
|  | 2002 year | 1.092 | 0.000 | 0.833 | 0.107 |
|  | 2004 year | 1.316 | 0.000 | 1.650 | 0.059 |
|  | 2005 year | 2.193 | 0.000 | 2.698 | 0.071 |
|  | 2006 year | 2.430 | 0.000 | 2.605 | 0.213 |
|  | 2007 year | 2.318 | 0.000 | 2.393 | 0.165 |
| 2004 year | 2001 year | 1.531 | 0.000 | 2.525 | 0.036 |
|  | 2002 year | 1.483 | 0.000 | 2.159 | 0.040 |
|  | 2003 year | 1.316 | 0.000 | 1.650 | 0.059 |
|  | 2005 year | 2.181 | 0.000 | 1.585 | 0.285 |
|  | 2006 year | 2.510 | 0.004 | 1.965 | 0.834 |
|  | 2007 year | 2.438 | 0.012 | 1.838 | 0.885 |
| 2005 year | 2001 year | 1.854 | 0.000 | 3.488 | 0.047 |
|  | 2002 year | 1.736 | 0.000 | 3.309 | 0.064 |
|  | 2003 year | 2.193 | 0.000 | 2.698 | 0.071 |
|  | 2004 year | 2.181 | 0.000 | 1.585 | 0.285 |
|  | 2006 year | 0.806 | 0.112 | 1.334 | 0.137 |
|  | 2007 year | 0.962 | 0.012 | 1.462 | 0.194 |
| 2006 year | 2001 year | 1.938 | 0.000 | 3.510 | 0.119 |
|  | 2002 year | 1.914 | 0.000 | 3.343 | 0.170 |
|  | 2003 year | 2.430 | 0.000 | 2.605 | 0.213 |
|  | 2004 year | 2.510 | 0.004 | 1.965 | 0.834 |
|  | 2005 year | 0.806 | 0.112 | 1.334 | 0.137 |
|  | 2007 year | 0.536 | 0.024 | 0.383 | 0.706 |
| 2007 year | 2001 year | 1.846 | 0.000 | 3.304 | 0.095 |
|  | 2002 year | 1.807 | 0.000 | 3.129 | 0.137 |
|  | 2003 year | 2.318 | 0.000 | 2.393 | 0.165 |
|  | 2004 year | 2.438 | 0.012 | 1.838 | 0.885 |
|  | 2005 year | 0.962 | 0.012 | 1.462 | 0.194 |
|  | 2006 year | 0.536 | 0.024 | 0.383 | 0.706 |

|  | | **WB1+2** | | **WB2** | | | **WB1** | |
| --- | --- | --- | --- | --- | --- | --- | --- | --- |
| **Time I** | **Time J** | **S.E** | **p-value** | | **S.E** | **p-value** | **S.E** | **p-value** |
| 2001 year | 2002 year | 2.001 | 0.185 | | 0.777 | 0.826 | 1.462 | 0.657 |
|  | 2003 year | 2.703 | 0.047 | | 1.934 | 0.054 | 0.468 | 0.086 |
|  | 2004 year | 3.247 | 0.003 | | 2.852 | 0.003 | 0.004 | 0.000 |
|  | 2005 year | 3.619 | 0.002 | | 2.779 | 0.000 | 6.023 | 0.390 |
|  | 2006 year | 2.502 | 0.001 | | 2.952 | 0.000 | 5.967 | 0.422 |
|  | 2007 year | 1.814 | 0.000 | | 3.029 | 0.000 | 5.391 | 0.373 |
| 2002 year | 2001 year | 2.001 | 0.185 | | 0.777 | 0.826 | 1.462 | 0.657 |
|  | 2003 year | 2.345 | 0.006 | | 1.378 | 0.011 | 1.930 | 0.411 |
|  | 2004 year | 2.317 | 0.000 | | 2.800 | 0.002 | 1.466 | 0.144 |
|  | 2005 year | 3.325 | 0.000 | | 2.930 | 0.000 | 7.485 | 0.491 |
|  | 2006 year | 3.104 | 0.001 | | 3.107 | 0.000 | 7.428 | 0.529 |
|  | 2007 year | 2.498 | 0.000 | | 3.155 | 0.000 | 6.853 | 0.482 |
| 2003 year | 2001 year | 2.703 | 0.047 | | 1.934 | 0.054 | 0.468 | 0.086 |
|  | 2002 year | 2.345 | 0.006 | | 1.378 | 0.011 | 1.930 | 0.411 |
|  | 2004 year | 2.182 | 0.007 | | 2.301 | 0.010 | 0.464 | 0.077 |
|  | 2005 year | 3.322 | 0.007 | | 3.449 | 0.000 | 5.555 | 0.524 |
|  | 2006 year | 3.199 | 0.028 | | 3.791 | 0.000 | 5.498 | 0.583 |
|  | 2007 year | 2.673 | 0.026 | | 3.748 | 0.001 | 4.923 | 0.515 |
| 2004 year | 2001 year | 3.247 | 0.003 | | 2.852 | 0.003 | 0.004 | 0.000 |
|  | 2002 year | 2.317 | 0.000 | | 2.800 | 0.002 | 1.466 | 0.144 |
|  | 2003 year | 2.182 | 0.007 | | 2.301 | 0.010 | 0.464 | 0.077 |
|  | 2005 year | 3.150 | 0.210 | | 4.472 | 0.021 | 6.019 | 0.861 |
|  | 2006 year | 3.556 | 0.905 | | 4.932 | 0.023 | 5.963 | 0.957 |
|  | 2007 year | 3.133 | 0.775 | | 4.774 | 0.027 | 5.387 | 0.897 |
| 2005 year | 2001 year | 3.619 | 0.002 | | 2.779 | 0.000 | 6.023 | 0.390 |
|  | 2002 year | 3.325 | 0.000 | | 2.930 | 0.000 | 7.485 | 0.491 |
|  | 2003 year | 3.322 | 0.007 | | 3.449 | 0.000 | 5.555 | 0.524 |
|  | 2004 year | 3.150 | 0.210 | | 4.472 | 0.021 | 6.019 | 0.861 |
|  | 2006 year | 1.961 | 0.045 | | 0.663 | 0.148 | 0.056 | 0.038 |
|  | 2007 year | 2.443 | 0.071 | | 0.638 | 0.901 | 0.632 | 0.598 |
| 2006 year | 2001 year | 2.502 | 0.001 | | 2.952 | 0.000 | 5.967 | 0.422 |
|  | 2002 year | 3.104 | 0.001 | | 3.107 | 0.000 | 7.428 | 0.529 |
|  | 2003 year | 3.199 | 0.028 | | 3.791 | 0.000 | 5.498 | 0.583 |
|  | 2004 year | 3.556 | 0.905 | | 4.932 | 0.023 | 5.963 | 0.957 |
|  | 2005 year | 1.961 | 0.045 | | 0.663 | 0.148 | 0.056 | 0.038 |
|  | 2007 year | 1.200 | 0.294 | | 2.550 | 0.045 | 0.576 | 0.564 |
| 2007 year | 2001 year | 1.814 | 0.000 | | 3.029 | 0.000 | 5.391 | 0.373 |
|  | 2002 year | 2.498 | 0.000 | | 3.155 | 0.000 | 6.853 | 0.482 |
|  | 2003 year | 2.673 | 0.026 | | 3.748 | 0.001 | 4.923 | 0.515 |
|  | 2004 year | 3.133 | 0.775 | | 4.774 | 0.027 | 5.387 | 0.897 |
|  | 2005 year | 2.443 | 0.071 | | 0.638 | 0.901 | 0.632 | 0.598 |
|  | 2006 year | 1.200 | 0.294 | | 2.550 | 0.045 | 0.576 | 0.564 |

**References**

1. Dye C, Floyd K. Tuberculosis, in Disease control priorities in developing countries, DT Jamison, et al., Editors. 2006, Oxford University Press: Washington D.C. pp. 289-309.
2. Ministry of Civil Affairs. [Brochure](http://www.njsaves.org/njswfile/America Saves-English Brochure.pdf) of administrative divisions of People’s Republic of China. Chinese society press, 2009.
3. Liu J. Achievements and Challenges of poverty alleviation in new era—interim evaluating report for Outline for Poverty Alleviation and Development of China's Rural Areas 2001-2010. China Financial & Economic Publishing House. 2006.
4. Gini, C. Measurement of Inequality and Incomes. The Economic Journal 1921; 31: 124–26.
5. [Wang L](http://www.ncbi.nlm.nih.gov/pubmed?term="Wang L"%5BAuthor%5D&itool=EntrezSystem2.PEntrez.Pubmed.Pubmed_ResultsPanel.Pubmed_RVAbstract), [Liu J](http://www.ncbi.nlm.nih.gov/pubmed?term="Liu J"%5BAuthor%5D&itool=EntrezSystem2.PEntrez.Pubmed.Pubmed_ResultsPanel.Pubmed_RVAbstract), [Chin DP](http://www.ncbi.nlm.nih.gov/pubmed?term="Chin DP"%5BAuthor%5D&itool=EntrezSystem2.PEntrez.Pubmed.Pubmed_ResultsPanel.Pubmed_RVAbstract). Progress in tuberculosis control and the evolving public-health system in China. Lancet. 2007; 369:691-6.
6. National Bureau of Statistics of China. China Statistical Year Book 2002. China Statistics Press, 2002-2008.
7. Bureau of Health Standards, Health Supervision, and Ministry of Health P.R. China. Collection of diagnostic standard and correlative laws and regulations for infectious disease. Beijing, BJ: China Standard Press, 2003.
8. Moran PAP. Notes on continuous stochastic phenomena, Biometrika 1950; 37: 17-23.
9. Getis A, Ord JK. The Analysis of Spatial Association by Use of Distance Statistics. Geographical Analysis 1992; 24:189-206.
10. Dye C, Zhao FZ, Scheele S, et. al. Evaluating the impact of tuberculosis control: number of deaths prevented by short-course chemotherapy in China. International Journal of Epidemiology 2000; 29: 558-64.
11. China Tuberculosis Control Collaboration. The effect of tuberculosis control in China. Lancet 2004; 364:417-22.
12. World Health Organization. Global Tuberculosis Control 2009: Epidemiology, strategy, finances. Geneva: World Health Organization, 2009.
13. Dong KY. Medical insurance system evolution in China. China Economic Review 2009; 20: 591-97.
14. Chen Z. New rural cooperative medical service will cover around whole country by 2008.[EB]．Xinhua，2007．09-05.
